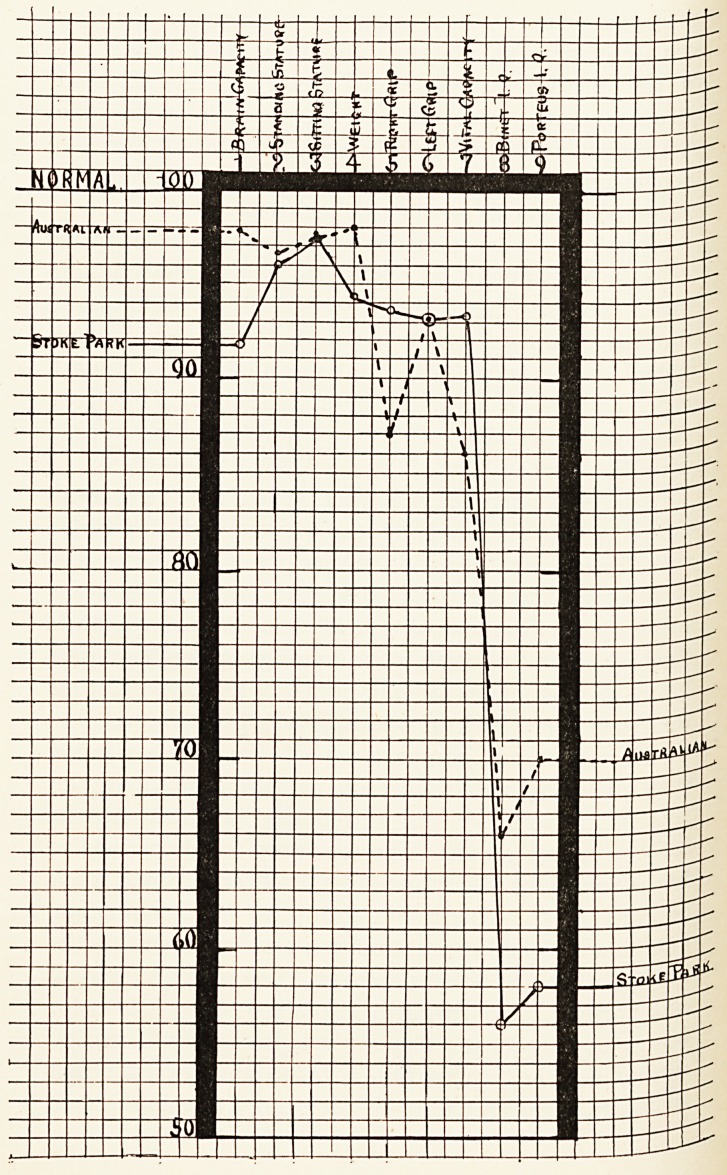# Mental Deficiency

**Published:** 1932

**Authors:** Richard J. A. Berry

**Affiliations:** Director of Medical Services, Stoke Park Colony, Stapleton, Bristol


### The Bristol Medico-Chirurgical Journal

" *Scire est nescire, nisi id me*
*Scire alius sciret.*"

AUTUMN, 1932.

### MENTAL DEFICIENCY:

### AN ANALYSIS OF THE MENTAL, PHYSICAL AND MEDICAL CHARACTERISTICS OF A GROUP OF ONE HUNDRED AND SIXTY-TWO ADULT FEEBLE-MINDED WOMEN.

BY

Richard J. A. Berry, M.D., F.R.C.S., F.R.S.E.,
*Director of Medical Services, Stoke Park Colony, Stapleton, Bristol.*

---

The medical staff of this Institution has recently conducted a co-operative examination of a group of 162 adult feeble-minded female inmates of the Colony. The investigation was not undertaken to further any preconceived opinions or theories as to the origin, causation and mental manifestations of mental deficiency, but simply to ascertain, first the facts, and second—if and where possible—the presence of any causative factor for the mental condition removable by medical care and treatment. For the

OCT 27 1932

first objective it was necessary to obtain a sufficiently large and homogeneous group to justify, statistically, any conclusions to be drawn for present or future comparative purposes. It was, therefore, proposed to examine 200 females of over 21 years of age and with a mental ratio of not less than seven years. But notwithstanding this relatively low mental standard and the large numbers of inmates of the Institution, it was not found possible to obtain more than 162 who satisfied the dual conditions, and of these some died, some were transferred to other institutions, and others were discharged on licence before the whole investigation could be completed; consequently the actual numbers vary slightly in some of the observations to be recorded.

The several lines of the investigation and those primarily responsible for their conduct were as follows :—

Physical and mental characteristics : the Director of Medical Services and the Psychological Staff.

Medical examination by Dr. J. A. Nixon, Professor of Medicine in the University of Bristol.

Neurological examination by Dr. R. G. Gordon.

Examination of the eye by Mr. A. E. Iles, F.R.C.S.

Examination of ear, nose and throat by Mr. Angell James, F.R.C.S.

X-ray examination of the skull by Dr. T. B. Wansbrough.

Some psychological experiments dealing with language ability, appreciation of spatial relationships and types of imagery by Dr. R. M. Norman in conjunction with Dr. R. G. Gordon.

Observations on the general behaviour and social reactions by the Matrons in charge of the cases.

1. *Physical and Mental Characteristics.*

The measurements and mental tests recorded fall into four chief groups :—

Head measurement and the calculation therefrom of cubic capacity of brain.

Physical measurements of standing and sitting stature and weight.

Neuro-muscular measurements of right and left grip and vital capacity.

Binet and Porteus tests.

As a full account of the objectives of the above, as well as of the mode of recording them has been published elsewhere,[1] the information need not be repeated. It is, however, of some interest to note that closely similar methods have been utilized by Doll in the United States,[2] by Dr. H. L. Gordon in Kenya Colony,[3] by Dr. Vint in Kenya Colony,[9] and by myself for a large group of Australian hospital cases.[4] The results for these Stoke Park defectives, which were worked out statistically and mathematically under my supervision by my assistants, Miss West and Miss Bergman, are as follows :—

| | N. | True mean. | Standard deviation. | Nearest normal age. |
|---|---|---|---|---|
| Head length .. | 162 | 177·35 ± 0·35 | 6·71 ± 0·25 | = 10·0 years. |
| Head breadth .. | 162 | 140·32 ± 0·29 | 5·58 ± 0·20 | = 10·0 years. |
| Head height .. | 162 | 121·85 ± 0·34 | 6·37 ± 0·23 | = 4·2 years. |
| Cubic capacity .. | 162 | 1215·33 ± 4·90 | 92·43 ± 3·46 | = 8·9 years. |
| Standing stature | 162 | 1537·88 ± 3·44 | 64·89 ± 2·43 | = 14·7 years. |
| Sitting stature .. | 162 | 830·94 ± 1·87 | 35·42 ± 1·32 | = 15·0 years. |
| Weights .. .. | 162 | 48·91 ± 0·37 | 7·09 ± 0·26 | = 16 years. |
| Right grip .. | 162 | 28·60 ± 0·29 | 5·56 ± 0·20 | = 16·6 years. |
| Left grip .. .. | 159 | 27·08 ± 0·29 | 5·47 ± 0·21 | = 17·0 years. |
| Vital capacity .. | 162 | 2282·41 ± 29·9 | 563·9 ± 21·1 | = 16·3 years. |
| Binet I.Q. .. | 162 | 55·66 ± 0·53 | 10·09 ± 0·37 | = 9·0 years. |
| Porteus I.Q. .. | 162 | 58·09 ± 1·11 | 21·01 ± 0·78 | = 9·4 years. |

True mean of the chronological age was 23·11 years.

Compared with normal children this group of adult defectives, with a true mean of 23·11 years, stands as follows :—

Size of head and brain equals that of a normal girl of 8 years 9 months.

The Binet mental ratio is that of a normal girl of 9 years.

The Porteus mental ratio is that of a normal girl of 9 years 4 months.

Physical development is that of a normal girl of 15 years 2 months.

Neuro-muscular development equals that of a normal girl of 16 years 7 months.

Assuming 18 years of age to express adult development — though such an assumption flatters the defectives—the years of their physical and mental retardation are as follows :—

For brain size, 9 years 3 months of retardation.

For mental ratio, 8 years 10 months of retardation; but if 16 years be taken to represent normality, then only 6 years 10 months of retardation.

For physical development, 2 years 10 months of retardation.

For neuro-muscular development, 1 year 5 months of retardation.

Differently expressed, these defectives are under-developed to greater or less degree on every count and measurement.

As regards size of head, 83·4 per cent. are so small-headed as to be definitely microcephalic, 0·5 per cent. are macrocephalic, and the remainder fall within the range of variability of normality. Of the microcephalic, 62 cases, or 38 per cent., were indeed so small-headed as to fall far below any previously recorded figure of brain capacity of normals. As four out of every five are thus definitely small-headed, it is not an unreasonable deduction to regard head measurement, recorded in accordance with the recommendations of the British Association for the Advancement of Science,[5] as a distinct advance in the scientific diagnosis of mental deficiency.

Further, if this tendency of the mentally defective to be microcephalic be read into, and in conjunction with, modern knowledge[6, 7] of cortical lamination and its significance it is again both reasonable and logical to assume the microcephaly of mental deficiency to be an index of a deficiency of cortical neurons, especially of the pyramidal-celled supra-granular cortex.

That this inference is correct, at least in the present series, is corroborated by the poverty of the Binet and Porteus mental ratios, which give a combined age of 9 years 2 months as compared with the 8 years 9 months for brain capacity. All three lines of approach thus agree in suggesting a seriously under-developed brain as the cause of the mental deficiency rather than any mere lack of educational opportunity.

These general comparisons of the feeble-minded with normals, as also of the present group with an Australian series,[4] are graphically set out in the accompanying graph. The tendency for defectives to be retarded, both physically and mentally, is as obvious in the Australian group as in the English, and thus the one series confirms and corroborates the other. Certain differences will, however, be noted. The former gives readings superior to the latter for brain capacity, physical development and mental ratios, and the explanation is to be sought in the fact that the English series is definitely feeble-minded,

1 Brain Capacity

2 Standing Stature

3 Sitting Stature

4 Weight

5 Right Grip

6 Left Grip

7 Vital Capacity

8 Binet I. Q.

9 Porteus I. Q.

NORMAL

100

Australian

Stoke Park

90

80

70

60

50

Australian

Stoke Park whereas the Australian group comprised patients of the Melbourne Children's Hospital sent down for examination and report by the Consultant Medical Staff to the Psychological Clinic then under my care. It was thus a mixed group of normals and defectives. On the other hand, the higher reading for the vital capacity of the English defectives is probably due to factors of age—adults in the one case and children in the other. Lastly, the Australian cases were of school age, and the English were beyond it. It is, however, of considerable interest to note that the higher mental ratios of the Australian group correlate with a better brain capacity, as the following figures, where normality is represented by 100, show :—

| | *Normal.* | *English. defectives.* | *Australian mixed group.* |
|---|---|---|---|
| Brain capacity | 100 | 91·8 | 97·8 |
| Binet I.Q. | 100 | 56·0 | 66·0 |
| Porteus I.Q. | 100 | 58·0 | 70·0 |

*Binet Mental Ratios.*—In view of the poor intelligence quotients recorded by this Stoke Park group of adult defectives with a true mean of only 55·66±0·53, it was deemed desirable to examine rather closely their performances with these tests. The Leland-Stanford revision comprises some eighty odd tests spread over, and allocated to, the years of life from the third to superior adult levels, and it was hoped that an analysis of the results might reveal the particular mental qualities lacking in these defectives, or possibly any special abilities they might possess. The main results of this analysis were as follows :—

*Fourth year tests.*—Four of our adult defectives had to be taken right back to the four-year-old level, and even then three failed to repeat four digits.

*Fifth year tests.*—These tests were attempted by 13 defectives, two of whom were unable to state their own age, and 23 per cent. were unable to perform three simple commissions.

*Sixth year tests.*—Attempted by 38 defectives, of whom 23 per cent. could not distinguish between right and left, and 42 per cent. failed to repeat simple syllables.

*Seventh year tests.*—Attempted by 89 defectives, 45 per cent. of whom were unable to repeat five figures and 39 per cent. failed to copy a diamond.

*Eighth year tests.*—Attempted by 135 defectives, of whom 34 per cent. were unable to count backwards from twenty, 39 per cent. could not comprehend simple problems of behaviour, and 35 per cent. were unable to give similarities between common, everyday objects.

*Ninth year tests.*—Attempted by 145 defectives, of whom, significantly enough, 65 per cent. failed to arrange five weights in sequence, thus suggesting a defective stereognostic sense, 54 per cent. were unable to give the correct change for simple money sums, 61 per cent. failed to repeat four figures backwards, and 59 per cent. were unable to find rhymes for simple words like day, mill and spring.

*Tenth and upper year tests.*—As all these, even where attempted, simply show a continuous series of failures it is not necessary to adduce further results.

Although the foregoing indicate only the more gross failures, it seems evident therefrom that the mental disability of these defectives is social rather than educational. When a group of adult young women record 30 per cent. to 50 per cent. failures at the performance of elementary commissions capable of being undertaken by a normal school child, are unable to comprehend the simplest problems of behaviour, cannot give the correct change for the simple monetary problems of everyday life, and display no single sign of ability at anything at all, it seems impossible to escape the logical deduction that they are socially defective, and that no amount of education of the ordinary scholastic kind can, or ever could have, overcome the natural and inherent defects of an immature and under-developed brain such as all the results go to prove these defectives possessed. The public misfortune appears to be that this social and cerebral disability is not appreciated soon enough, and hence much time and even more money are wasted in attempting, educationally, to "make silk purses out of sows' ears." Defectives of this kind can, therefore, only find a place in the social life of any community, institutional or otherwise, as "hewers of wood and drawers of water," and in this capacity some of them might even find their level of social efficiency.

*Social Reactions and General Behaviour.* — The observations of the Matrons in charge on the social reactions and general behaviour of their charges are best introduced here, as they will afford a personal corroboration or otherwise of the results already adduced. From their daily contact with the inmates the Matrons are obviously in an exceptional position to furnish such information. On the other hand, it is to be remembered that the reactions of a segregated defective, as regards personal honesty and sexual morality, necessarily differ from those at large in the community. The opportunities for the former are disciplined and few in number. Those for the latter are neither one nor the other.

The number of defectives to whom the foregoing apply were, in the aggregate, 158. Of these 90 per cent. are reported to be unstable in conduct and behaviour; 38 per cent. are quarrelsome, disobedient and devoid of all sense of discipline; 15 per cent. are dishonest, immodest and lacking in affection. In addition to these leading characteristics many other social peculiarities were mentioned by the officers in charge. Amongst these are "viciously immoral," "immoral tendencies," "childish," "uncontrolled emotional reactions, always weeping or always crying," "bad language, violent and destructive," "resents correction and strikes officers," "silly and moody," and so on through the whole range of those human aberrations of social behaviour, which so clearly reveal man's kinship to the animal kingdom.

That all these several studies of head form, undeveloped bodies and brains, animal behaviour, lack of intelligence and control, mental stupidity and the like, should agree in pointing to the common factor at fault, namely, an undeveloped brain lacking in that refinement of structure so essential to a human mind, will surprise no one with first-hand personal knowledge of mental defectives and their ways. What does cause surprise is that anyone should fail to realize that these results so entirely agree with and support those previously made by Shaw Bolton, Mott, Watson, Tredgold, and others[8] on the meaning and significance of cortical lamination with its supra- and infra-granular cortices. Clearly these defectives have an inefficiently developed supra-granular pyramidal-celled cortex, and are thus unable to control effectively those more animal instincts and reactions mediated, according to Watson, by the polymorphic-celled infra-granular cortex. A deep-seated acquaintance with modern cortical physiology would appear to be the key to further progress in the study of mental deficiency.

2. *Neurological Examination.*

A neurological examination of 158 of these defectives was conducted by Dr. R. G. Gordon. The main objective was the determination of the presence or absence of nervous diseases, apart from the mental condition, either as a cause or consequence of the amentia.

The general result of this examination shows that, while there are no clinical syndromes at all pathognomonic of mental deficiency, the condition itself appears to be associated with many forms of nerve disorders, thus suggesting the possibility that the under-development so manifest in the brain involves all parts of the nervous system in greater or less degree.

*Absence of Clinical Syndromes.*—Only five cases showed recognizable clinical syndromes, namely three hemiplegias, one paraplegia, and one case of encephalitis Mental deficiency does not, therefore, appear to be either the result or consequence of serious nervous disease.

*Presentation of the Nerve Phenomena Observed.*—In view of the absence of recognizable nervous diseases in this group of cases it may be instructive to present the phenomena observed in accordance with the general principles underlying the nervous system, which may be briefly stated as follows :—

"All the phenomena presented by the functioning of the nervous system, normal and abnormal, fall into four great groups : absence, diminution, perversion or exaggeration, of the functions of the three great structural divisions of the nervous system, receptor, internuncial and effector neurons."[1]

*The Receptor Side.*—As regards proprioceptive sensibility as indicated by a sense of posture in feet and hands, and exteroceptive sensibility, as tested by the patient's ability to localize points of contact on the skin, where an error of more than 4 cm. was regarded as abnormal, and by the ability to discriminate between contact of one or two points on the skin, it is instructive to note that 117 cases (74 per cent.) showed a diminution or defect of such sensibilities, 13 patients displayed a diminished sensibility to pain, and 2 an exaggerated sensibility to such. These results can, from a structural consideration, only indicate one or other, or both, of two factors: a reduction in the numbers of those centrally conducting receptor axons whose cell stations are in the dorsal nerve root ganglia, or a reduction in the numbers and quality of the receptive cortical neurons, believed by Kappers and others to be the granulous Golgi type II cells of the cortex. This last factor would, of course, necessarily denote a diminution in the sensibilities tested, and the observation thus falls into line with the previously recorded results.

*The Cortical Internuncial and Other Tissues.*—This tissue comprises practically all of the cortex exclusive of the well-established receptor and effector pathways to and from the brain. The abnormalities attributed, in this series of cases, to this tissue fall into three groups: abnormalities of speech, abnormalities of emotional stability and control, clinical signs of psychoses, and other disordered reactions.

Sixteen cases (10·1 per cent.) displayed abnormalities of speech such as lisping, slurring, monotone speech and staccato utterance. Of these one was hemiplegic.

Thirty-six cases (22·7 per cent.) showed "temper tantrums," three of these being described as violent, whilst one was associated with epilepsy. On the other hand, the matron in charge of the cases and in daily contact with them reported ninety-seven cases (61·4 per cent.) as being bad-tempered.

Four cases showed psychotic symptoms. One had delusions of pregnancy, one of persecution, one displayed auditory hallucinations, and one—one of the lowest grades of the lot—soiled the linen cupboard with fæces.

Abnormalities of cerebral neuronic arcs also displayed themselves in the forms of exaggerated or diminished movements, chiefly the former. Amongst these were five cases (0·3 per cent.) with epileptic convulsions (grand mal). Thirty cases (18·9 per cent.) displayed choreic movements, fifty cases (31·6 per cent.) suffered from fine and coarse tremors (forty-four and six cases respectively), and one case suffered from spasmodic torticollis with other exaggerated movements.

*The Effector Side.*—On the other hand, twenty-seven cases (17·1 per cent.) showed a diminution of muscle tonus as displayed by diminished tendon reflexes. Sixty-three cases (39·9 per cent.) showed definite alterations in pyramidal or effector pathway activity, chiefly in the direction of exaggerated reflexes, and of these ten had apparently suffered from congenital syphilis.

Whilst it is not easy to elicit from institutional life authentic evidence of sex manifestations or aberrations, unless they are particularly obtrusive, six of this group appeared to be definitely homo-sexual.

From his much more detailed analysis of these 158 feeble-minded patients Dr. Gordon states that "while abnormalities are distributed all through the nervous systems of these aments, very few show signs or symptoms of definite clinical syndromes." He therefore concludes that "mental deficiency, even in the higher grades, is associated with an imperfectly-developed nervous system, especially of the cerebral cortex"—a conclusion which is thus in strict and independent association with the rest of the research.

3. *General Medical Examination.*

Of all the available cases of this group a thorough and complete general medical examination was carried out by Dr. J. A. Nixon, Professor of Medicine in the University of Bristol and Consulting Physician to this Colony. The objectives of this part of the investigation were the determination of general physical disease as a possible causative factor of mental deficiency, the therapeutic treatment of any disease to which the individual mental deficiency might reasonably be attributed, and the collection of any medical scientific data pointing to the association of any particular type of disease with cerebral amentia.

With these objectives an exhaustive medical examination was made of the various systems of the body, cardio - vascular, respiratory, genito - urinary, etc., and from the general results obtained from this examination it is evident that there is no particular physical medical ailment specially associated with mental deficiency, either as a causative agency or as a usual concomitant, and that mental deficiency is in itself neither a manifestation of disease nor of a complex of diseases. On the contrary, this medical examination again stresses the fact, as do all the other lines of the research, that it is to a lack of development that the physical, medical and mental phenomena are due, rather than to disease as such.

All the ordinary medical ailments prevalent in any general mentally normal population presented themselves amongst this group of high-grade defectives, and did so in about the same general proportions. Syphilis was naturally looked for systematically, and the Wassermann reaction taken in every case. Congenital syphilis was detected clinically in nineteen cases—that is, in 10·1 per cent.—fifteen of which gave a positive Wassermann reaction, and Hutchinson's teeth were present in three cases. The urine was systematically and completely examined in 119 cases, 88 of which showed no abnormality or sign of disease apart from a very high urobilin content. B. coli bacilluria, without any symptoms, was found in five cases, and marked oxaluria in twenty-five cases. Apart from the fact that the diet of these patients is generous as regards vegetables no adequate explanation of this frequent occurrence of oxaluria is forthcoming, whilst the extraordinarily high urobilin content, even up to 140 times the normal, seems to have no clinical significance, whatever it may have physiologically.

The general deduction from this medical examination is, then, that there is no disease specially associated with mental deficiency other than those of congenital—that is, developmental—origin, as is shown by the high incidence of congenital heart disease amongst these defectives. In some of these cases the heart affection was associated with presumable causal factors such as rheumatism (three cases) or syphilis (seven cases), but in twenty-two cases—or 18·5 per cent.—no such obvious causative factor was discoverable, and hence it seems reasonable to infer that the cardiac lesion was developmental in origin. Amongst the school children of Bristol Dr. Bruce Perry found only 116 cases of congenital heart disease in a school population of 55,000, that is 0·2 per cent. in a normal population as against 18·5 per cent. in a mentally defective one.

4. *Specialist's Examination of the Receptor Organs of Sight and Hearing.*

In view of the very great importance in the development of the normal brain cortex of incoming exteroceptive stimuli of light and sound waves and the serious results which may accrue from complete or partial deprivation of such senses, it was clearly of importance to submit these defectives to expert examination as regards the eye and the ear. The physiological principle underlying this part of the examination is, of course, that if the receptor organs of sight and hearing, namely the retina and the organ of Corti, are defective or seriously impaired there must be a corresponding diminution of stimuli conveyed to the appropriate cortical areas, with a consequent impairment of development of the brain cells themselves, and a resultant greater or less degree of amentia.

A careful examination of the eyes of 155 of these cases was carried out by Mr. A. E. Iles, F.R.C.S., Consulting Ophthalmologist to the Institution. He states that the chief departure from the normal appeared in these defectives to be the greater incidence of congenital errors of development, as compared with what he himself found amongst 7,721 cases of out-patients at the Bristol Eye Hospital. Thus, amongst these 155 defectives there were eleven cases of congenital cataract—that is, 7·1 per cent. as against 0·5 per cent. in the general population. Other results, with their hospital incidence, may be set forth as follows :—

| | 155 *Stoke Park Defectives.* | 7,721 *Hospital Cases.* |
|---|---|---|
| Congenital ptosis .. | 1 case, 0·64 per cent. | 9 cases, 0·1 per cent. |
| Convergent strabismus | 10 cases, 6·4 per cent. | 308 cases, 3·9 per cent. |
| Divergent strabismus | 8 cases, 5·2 per cent. | 34 cases, 0·4 per cent. |
| Nystagmus .. .. | 9 cases, 5·8 per cent. | 19 cases, 0·2 per cent. |
| Persistent pupillary membrane .. .. | 5 cases, 3·2 per cent. | 2 cases, 0·2 per cent. |
| Persistent hyaloid arteries .. .. .. | 2 cases, 1·3 per cent. | 1 case. |
| Coloboma of choroid | 1 case, 0·6 per cent. | 3 cases, 0·3 per cent. |

As regards errors of refraction, these were very common, and many of them gave an error of more than 1D of either hypermetropia or myopia. In fact, there were more errors above 1D error of refraction than there were less than this, and in addition 35 cases out of 155 (22·5 per cent.) had errors of more than +3 or –3D, and there were four cases (0·2 per cent.) greater than +6 and six cases (0·3 per cent.) more than –6.

In addition to the above congenital or developmental errors other eye conditions were noted. Amongst these were two cases of optic atrophy, two cases of optic neuritis, one of retinitis pigmentosa, one of atresia of the puncta, twenty cases in which the chorio-capillary layer was very definitely deficient, whilst old interstitial keratitis was found in five cases (3·2 per cent. as against 0·4 per cent. in Mr. Iles's hospital population).

Mr. Iles concludes that his examination of the eyes shows a decided fault in development in these high-grade defectives. In twelve cases even the best correction only gave vision less than 6/60, suggesting, of course, a lack of cortical visual neurons. On the other hand, there were few cases of colour-blindness; in fact, only one was definitely truly colour-blind, but a few had defective yellow-green vision. The method employed for testing colour vision was Ishiwara's.

It thus appears from this important investigation into the organ of vision as a receptor avenue of approach to the brain that in defectives the eye shares with the rest of the body and brain a general lack of development, but that in the majority of these cases the eye condition cannot be held responsible for the amentia, and that even the best treatment for the former would not, in these cases, materially benefit or improve the latter.

5. *Examination of Ear, Nose and Throat.*

An examination was made by Mr. Angell James of 137 of these mentally defective females. The object of the investigation was to determine if there was any variation from the normal anatomical or physiological condition, chiefly of the ears, but also of the noses and throats of these individuals. The cases are divided into two groups as follows :—

(1) Eighty cases, who showed no signs of acquired disease of the ears.

(2) Fifty-seven cases, in which signs of disease were present.

No case of gross congenital deformity was found. In both groups the percentage of cases showing deviation of the septum was approximately the same, twenty-seven in Group 1 and twenty-three in Group 2. In the great majority it was obviously due to trauma, but the absence of a reliable history made it impossible to quote useful figures. Only in 3 per cent. was it sufficient to produce any local symptoms.

*Group 1.*—In this group signs of infection or other disease of the nose or throat were rare. Only one case of sinusitis was seen, and two cases of atrophic rhinitis. 6 per cent. had been operated on for removal of tonsils, and half of these showed septic remains. Of the remainder, 54 per cent. had perfectly healthy and clean tonsils. Only 5 per cent. of the other 46 per cent. showed any more than some collections of debris in the crypts and very slight evidence of inflammation. In 9 per cent. there was some enlargement of adenoids, one case having been operated upon, but in none was the enlargement causing local symptoms.

Of the hearing tests employed that for the hearing distance of a watch was found to be the most reliable for these cases.

Altogether 180 ears were tested. In 90 per cent. of cases the two ears gave equal results, 31 per cent. gave the normal average of 20 inches. The average hearing distance of all the cases, however, was only 11 inches. In 15 per cent. there was, in addition, slight reduction in the hearing time by air conduction for 32 and 256 [D.V.S.] tuning forks. All the other tests for bone conduction, upper tone limit, etc., gave normal results.

*Group 2.*—In this group there were 23 per cent. showing perception (nerve) deafness. The remaining 67 per cent. had some lesion of the conducting apparatus, 7 per cent. had active suppuration, and the remainder showed cicatricial and catarrhal changes ; 31 per cent. were unable to hear the watch, while the average hearing distance for all was 3 inches.

In this group 53 per cent. had perfectly healthy and clean tonsils, 16 per cent. had been operated upon, but half of these showed septic remains.

The figures show that although there were no cases of gross congenital deformity in these groups, in the group without any sign of acquired aural disease the average auditory acuity was slightly subnormal. But in no case was it so marked that any failure of development of the brain could be attributed to lack of stimulation via the auditory apparatus.

On the other hand, there was no evidence that any disease of these regions is associated with, or abnormally prevalent in, these groups. Although 41 per cent. may seem a large percentage for cases showing evidence of acquired aural disease, the standard set was very high. Even very doubtful evidence was considered sufficient to exclude the case from the normal group.

No association could be shown between mental deficiency and focal infection from these regions, or enlargement or infection of the tonsils and adenoids.

The subnormal auditory acuity is in accordance with the subnormal reaction of the nervous system in these cases.

6. *X-ray Examination of the Skull.*

For technical and clinical reasons this examination of the skull by Dr. T. B. Wansbrough has had to be postponed, but will be undertaken at a later date.

7. *Psychological Experiments dealing with Language Ability, etc.*

The results of these experiments were communicated by Drs. Gordon and Norman to the British Association for the Advancement of Science at the Centenary Meeting held in London in 1931. They are now being published in the *British Journal of Psychology*, vol. xxiii., 1932.

*Causes of Mental Deficiency as Revealed by this Series.*

In view of the differences of opinion as to the hereditary or acquired origin of mental deficiency the following analysis of the 162 cases may prove of interest.

Of the 158 cases examined by Dr. Gordon there were only three which gave a "clear and unequivocal history of either injury or infection exclusive of syphilis." In one case there was evidence of a severe injury to the frontal region of the head; of severe injury to the left parietal region in another, and a history of encephalitis in a third. Notwithstanding that such injuries do not definitely exclude hereditary transmission, these three cases may be tentatively accepted as of secondary origin.

Of the 162 cases comprising the series there was no previous history of any sort in twenty-five instances. Details of the parentage and familial relationships were available in the remaining 137 cases, with specific information in 55 cases of the presence or otherwise of mental disorder or deficiency in the parents or blood relations. In these 55 cases there was direct and unequivocal evidence of the hereditary origin of the condition in 46 cases, and strong presumptive evidence of the same in the remaining 9 cases.

The relative proportions of hereditarily transmitted, and of secondarily acquired, amentias thus work out in this series as follows :—

| | |
|---|---|
| Number of cases, 137 (hereditarily transmitted): | |
| Direct evidence .. .. | 46 cases=33·6 per cent. |
| Presumptive evidence .. | 9 cases= 6·6 per cent. |
| Insufficient evidence available .. .. | 82 cases=59·8 per cent. |
| Number of cases, 158 : | |
| Secondarily acquired .. | 3 cases= 0·2 per cent. |

In this series there can, then, be no doubt of the strong influence of heredity in the transmission and causation of mental deficiency.

### CONCLUSION.

This and other allied investigations into mental deficiency seem to suggest, if not indeed to prove, that mental deficiency is a manifestation of improper development and not of disease, so that the problem becomes one of preventive medicine, eugenics and embryology rather than of curative medicine. If, then, researches such as these should succeed in directing the attention of the profession and the lay public to the true causes of a great national problem, the labour and time devoted to them will not have been in vain.

REFERENCES.

[1] Berry, R. J. A., *Brain and Mind*, chapters 41, 42 and 43. New York: The Macmillan Co. 1928.

[2] Doll, E. A., *Anthropometry as an Aid to Mental Diagnosis*. Publications of the Training School, Vineland, New Jersey, No. 8, February, 1916.

[3] Gordon, H. L., " A Note on Diagnosis of Amentia (Mental Deficiency) in Africans," *The Kenya and East African Medical Journal*, vol. vii., No. 8, November, 1930, pp. 208 to 214.

[4] Berry, R. J. A., *Report to the Edward Wilson (Argus) Trust on Mental Deficiency in the State of Victoria, with Suggestions for the Establishment of a Child Guidance Clinic.* Published in and by the *Argus*, Melbourne, 1929.

[5] British Association for the Advancement of Science: *Anthropometric Investigation in the British Isles. Report of the Committee (being the Final Report). Reprinted, with additional illustrations, by permission of the Council from the Report of the British Association.* Dublin, 1908. The Royal Anthropological Institute, 1909.

[6] Berry, R. J. A., and Porteus, S. D., *Intelligence and Social Valuation. A Practical Method for the Diagnosis of Mental Deficiency and Other Forms of Social Inefficiency.* Publications of the Training School at Vineland, New Jersey, Department of Research, No. 20, May, 1920.

[7] Berry, R. J. A., " The Correlation of Recent Advances in Cerebral Structure and Function with Feeble-mindedness and its Diagnostic Applicability," *Medical Journal of Australia.* Supplement. (Transactions of Congress.) 7th June, 1924, pp. 393–400.

[8] Shaw Bolton, J., *The Brain in Health and Disease.* London: Edward Arnold. 1914

Shaw Bolton, J., " Recent Researches on Cortical Localization and on the Functions of the Cerebrum," article in Leonard Hill's *Further Advances in Physiology*, pp. 284–350. London: Edward Arnold. 1909.

Mott, Watson and Tredgold, various contributions in the *Archives of Neurology*, vols. ii., iii., iv. and v. London: Macmillan & Co. Ltd. 1903–1911.

[9] Vint, F. W., " A Preliminary Note on the Cell Content of the Prefrontal Cortex of the East African Native," *East African Medical Journal*, May, 1932.

## Figures and Tables

**Figure f1:**